# Comparing Polymerase Chain Reaction Testing of Nasopharyngeal Swab and Lower Respiratory Tract Specimens for the Diagnosis of *Pneumocystis jirovecii* Pneumonia

**DOI:** 10.1093/ofid/ofae071

**Published:** 2024-02-02

**Authors:** Rusheng Chew, Sarah Tozer, Kimberly Ulett, David L Paterson, David Whiley, Theo Sloots, David Fielding, Christopher Zappala, Farzad Bashirzadeh, Justin Hundloe, Cheryl Bletchley, Marion L Woods

**Affiliations:** Centre for Tropical Medicine and Global Health, University of Oxford, Oxford, United Kingdom; Mathematical and Economic Modelling Department, Mahidol Oxford Tropical Medicine Research Unit, Bangkok, Thailand; Faculty of Medicine, University of Queensland, Brisbane, Australia; Central Laboratory, Pathology Queensland, Central Laboratory, Brisbane, Australia; Department of Medicine, Gold Coast Hospital and Health Service, Gold Coast, Australia; Central Laboratory, Pathology Queensland, Central Laboratory, Brisbane, Australia; UQ Centre for Clinical Research, University of Queensland, Brisbane, Australia; Infectious Diseases Unit, Royal Brisbane and Women's Hospital, Brisbane, Australia; UQ Centre for Clinical Research, University of Queensland, Brisbane, Australia; Faculty of Medicine, University of Queensland, Brisbane, Australia; Faculty of Medicine, University of Queensland, Brisbane, Australia; Department of Thoracic Medicine, Royal Brisbane and Women's Hospital, Brisbane, Australia; Faculty of Medicine, University of Queensland, Brisbane, Australia; Department of Thoracic Medicine, Royal Brisbane and Women's Hospital, Brisbane, Australia; Department of Thoracic Medicine, Royal Brisbane and Women's Hospital, Brisbane, Australia; Department of Thoracic Medicine, Royal Brisbane and Women's Hospital, Brisbane, Australia; Central Laboratory, Pathology Queensland, Central Laboratory, Brisbane, Australia; Faculty of Medicine, University of Queensland, Brisbane, Australia; Infectious Diseases Unit, Royal Brisbane and Women's Hospital, Brisbane, Australia

**Keywords:** nasopharyngeal swab specimen, *Pneumocystis jirovecii* pneumonia, specimen quality, test performance

## Abstract

Using nasopharyngeal (NP) swab samples instead of lower respiratory tract specimens for polymerase chain reaction (PCR) to diagnose *Pneumocystis jirovecii* pneumonia (PJP) may be better tolerated and improve diagnostic accessibility. In this 2-year Australian retrospective cohort study of patients with clinically suspected PJP, *P jirovecii* PCR on NP swab samples had perfect specificity but low sensitivity (0.66).

The unicellular fungus *Pneumocystis jirovecii* is an opportunistic pathogen causing pneumonia in immunocompromised hosts [1], such as those with human immunodeficiency virus (HIV)/AIDS [2]. It is also the most common fungal cause of pneumonia in non–HIV-infected children <5 years old [3]. Despite global improvements in HIV prevention and treatment, the burden of *P jirovecii* pneumonia (PJP) remains considerable, owing to the increasing use of immunosuppressive agents for transplantation, cancer, and autoimmune disorders, along with suboptimal control of the HIV/AIDS epidemic in developing countries [4, 5].

Because *P jirovecii* is not culturable in vitro, polymerase chain reaction (PCR) is the reference standard diagnostic method in many countries [6, 7]. *P jirovecii* PCR is generally performed on lower respiratory tract specimens, such as induced sputum or bronchoalveolar lavage fluid specimens. However, patients may be too frail, young, or hypoxic to undergo such procedures, especially fiberoptic bronchoscopy, which may result in unintended morbidity or death [8]. Lower respiratory tract specimen collection may be invasive and carries a risk of patient discomfort, such as bronchospasm from sputum induction [9]. It also requires trained staff, as well as costly equipment and facilities, such as negative pressure rooms, resulting in service inequity for patients in rural or remote locations and developing countries [7, 8].

There is therefore a need for specimens that can be obtained using low-cost, minimally invasive methods and on which *P jirovecii* PCR can be performed, without compromising test performance. Nasopharyngeal (NP) swab specimens may be such an alternative. Nevertheless, to our knowledge at the time this study was conducted, the only evidence comparing NP and lower respiratory tract specimens came from children in a developing country with high HIV prevalence published more than a decade ago [10], indicating a gap in the contemporary evidence. In the current study, we aimed to evaluate *P jirovecii* PCR performed on NP swab specimens as a diagnostic test for PJP, as well as the impact of specimen quality on test performance.

## METHODS

### Study Design

Retrospective cohort study. The study STROBE checklist can be found in the Supplementary Materials.

### Setting and Participants

Queensland is an Australian state with an estimated HIV prevalence of 0.14% [11]. Eligible patients were those with clinically suspected PJP in public sector hospitals who had *P jirovecii* PCR performed on lower respiratory tract specimens (either induced sputum or bronchoalveolar lavage fluid) over 2 years from 1 January 2015 to 31 December 2016 and also had NP swab specimens collected by healthcare staff within 7 days of lower respiratory tract specimen collection. Patients were identified through a state-wide computerized database (AUSLAB; Citadel Health), which contains records of all laboratory investigations requested in public sector healthcare facilities and relevant supporting clinical details for each patient.

### PCR method

*P jirovecii* PCR was performed twice on each lower respiratory tract and NP swab specimen; a positive result for each specimen was returned if *P jirovecii* DNA was detected with either of the 2 tests.

The PCR detection and subsequent quantification of *P jirovecii* was performed using an in-house real-time method on a Rotor-Gene instrument (Qiagen). The mix was prepared using the Qiagen QuantiTect PCR kit (Qiagen ) incorporating specific primers and probe targeting the 5-second ribosomal RNA gene, details of which are as follows: forward primer (PCP-TM-5s-F), AGTTACGGCGATACCTCAGAGAATATAC; reverse primer (PCP-TM-5s-R), GCTACAGCACGTCGTATTCCCATA; and probe (PCP-TM-5s-probe), FAM-TCACCCACTATAGTACTGACGACGCCCTT-BHQ.

The control and quantitative standards were prepared in house using extracted patient material with high copy numbers. The standard for the qualitative assay was prepared at 2.5 × 10^10^ copies/mL, while 4 standards were prepared to provide a standard curve with the range 8.0 × 10^4^—8.0 × 10^7^ copies/mL for the quantitative assay. The standard curve was plotted with the supplied Rotor-Gene software using the quantitation option, and quantitative results for samples were determined from this plot by the software. Results were expressed as copies per milliliter.

### Assessment of Specimen Quality

Specimen quality was assessed with human DNA quantification, using endogenous retrovirus 3 (ERV3) as a surrogate marker, per the method of Alsaleh et al [12]. Because each diploid human cell contains 2 copies of this retrovirus, ERV3 PCR allows accurate quantification of the number of human cells present in a sample; the higher the ERV3 load, the better the quality of the specimen. Its use for this purpose is well established, with high analytical sensitivity and specificity [13].

### Statistical Analysis

Patients for whom NP swab specimen *P jirovecii* PCR results were unavailable were excluded from the analysis. Of the remaining patients, only those with nonmissing values for the ERV3 load were included in the assessment of specimen quality. Patients who tested negative for ERV3 (ie, had an ERV3 load of 0), were not considered to have missing values for ERV3 load. We calculated the diagnostic sensitivity and specificity of NP swab specimens for PJP, as well as the positive and negative predictive values, using results with lower respiratory tract specimens as the reference standard.

To assess the impact of specimen quality, ERV3 loads were compared as follows, using Mann-Whitney *U* tests: in lower respiratory tract specimens from patients with or without PJP (ie, true-positives and true-negatives), in NP swab specimens from true-positives and true-negatives, and in NP swab specimens from true-positives and false-negatives. Analyses were performed using Stata 17 software (StataCorp). Differences were considered significant at *P* ≤ .05. Raw data are available on request from the corresponding author.

### Patient Consent and Ethical Approval

This study does not include factors necessitating patient consent. Ethical approval was obtained from the Royal Brisbane and Women's Hospital Ethics Committee (HREC/14/QRBW/19).

## RESULTS

One hundred eleven patients met the inclusion criteria; their characteristics are summarized in Table 1. Specimen quality was assessed in 108 of 111 (97.3%) lower respiratory tract specimens and 98 of 111 (88.3%) NP swab specimens for which ERV3 load data was available. From the data in Table 1, the diagnostic sensitivity of NP swab specimen *P jirovecii* PCR was 0.66, while its specificity was 1.0. The positive predictive value was 1.0, and the negative predictive value, 0.63.

**Table 1.** Participant Characteristics

Characteristic	Patients, No. (%)^a^	
	With PJP (n = 71)	Without PJP (n = 40)
Age, median (IQR), y	66 (55–72)	62 (46–73)
Sex		
Male	32 (45.1)	19 (47.5)
Female	39 (54.9)	21 (52.5)
Immunosuppression type		
Hematological cancer or transplant	13 (18.3)	14 (35.0)
Solid organ cancer	28 (39.4)	7 (17.5)
Solid organ transplant	3 (4.2)	7 (17.5)
HIV positive status	11 (15.5)	1 (2.5)
Other iatrogenic immunosuppression	14 (19.7)	4 (10.0)
Other noniatrogenic immunosuppression	2 (2.8)	7 (17.5)
NP swab *Pneumocystis jirovecii* PCR result		
Negative	24 (33.8)	40 (100.0)
Positive	47 (66.2)	0 (0.0)

Abbreviations: HIV, human immunodeficiency virus; IQR, interquartile range; NP, nasopharyngeal; PCR, polymerase chain reaction; PJP, *P jirovecii* pneumonia.

^a^Data represent no. (%) of patients unless otherwise specified. Patients with *Pneumocystis jirovecii* pneumonia (PJP) were those who were clinically suspected of having the disease and had positive *P jirovecii* PCR results with lower respiratory tract specimens (induced sputum or bronchoalveolar lavage fluid).

The median ERV3 loads in lower respiratory tract specimens did not differ significantly between patients with true-positive and those with true-negative results (4.25 × 10^3^ vs 8.56 × 10^3^, respectively; *P* = .06). The median NP swab specimen ERV3 loads also did not differ significantly between these 2 groups (6.20 × 10^2^ vs 1.71 × 10^3^, respectively; *P* = .07). However, the median ERV3 load in NP swab specimens from true-positives was significantly higher than in those from false-negatives (7.55 × 10^2^ vs 3.67 × 10^2^, respectively; *P* = .0499).

## DISCUSSION

We found *P jirovecii* PCR of NP swab specimens to be highly specific (specificity, 1.0) but poorly sensitive (sensitivity, 0.66) for diagnosing PJP, compared with lower respiratory tract specimens in immunosuppressed adult patients living in a developed country with low HIV prevalence. Importantly, the positive predictive value was 1.0. Given the absence of false-positive results, this indicates that a positive PCR result with an NP swab specimen is sufficient for diagnosing PJP. It follows that our results suggest that colonization of the upper respiratory tract by *P jirovecii* is not a barrier to performing PCR on NP swab specimens for this purpose in adults.

We also showed that false-negative NP swab specimens contained significantly lower ERV3 loads than true-positive specimens, demonstrating the importance of proper specimen collection to ensure adequate quality and prevent misclassification [14]. One method of improving the chances of adequate specimen collection may be to collect >1 swab specimen for testing in parallel. This has the potential added benefit of increasing sensitivity, since sensitivity would be expected to increase to 88% with 2 swab specimens and to 96% with 3, although this has yet to be conclusively proved. Such augmentation of the performance of low-sensitivity assays with multiple testing has been demonstrated with rapid antigen tests for severe acute respiratory syndrome coronavirus 2 in a real-world population during the current coronavirus disease 2019 pandemic [15], although a potential barrier to this approach may be that *P jirovecii* does not necessarily migrate to the upper respiratory tract in all cases of PJP.

Our results align with those of Lieu et al [16], who also found that the specificity and sensitivity of *P jirovecii* PCR on NP swab specimens or aspirates was 1.0 and 0.6 when compared with paired lower respiratory tract specimens taken up to 7 days apart. Our finding of perfect specificity also agrees with the results reported by Sivaraj et al [17]. A key result of all 3 studies is that no false-positives were seen, again supporting the hypothesis that *P jirovecii* colonization of the upper respiratory tract may not necessarily preclude the use of PCR of NP swab specimens to diagnose PJP.

A key strength of our study is the finding that ERV3 loads did not differ significantly between true-positive and true-negative patients, for both NP swab and lower respiratory tract specimens. This indicates that the quality of specimen collection was equivalent for both specimen types in both groups, and it negates the possibility of differing specimen collection quality affecting the results. Another benefit of using NP swab specimens is the ability to test for concurrent or alternative diagnoses simultaneously, for example, with a multiplex PCR panel; in our cohort, 12 of 71 patients with and 6 of 40 without PJP also had a PCR-confirmed viral respiratory infection.

Our results are not generalizable to children, as none as featured in our data set. The number of patients in this study was small, which was a potential source of bias, as was the study’s observational nature, focused on patients with high pretest probabilities for PJP. Moreover, that results may depend on the sensitivity and specificity of the PCR platform used. A final limitation is the use of paired specimens collected up to 7 days apart; in this time period, treatment may have been commenced based on clinical suspicion, which could affect the fungal load and hence the result of the later test.

While we expect *P jirovecii* PCR on NP swab specimens to perform similarly in adult patients from other settings with low HIV prevalence, this hypothesis should be tested as part of future research. Ideally, this would be a prospective study, including all patients clinically suspected of having PJP in a wider range of settings and patient demographics, with paired lower respiratory tract specimens and 2–3 NP swab samples collected from each patient, using the correct technique on the same day.

In conclusion, our hypothesis-generating study demonstrates the potential utility of NP swab samples for the diagnosis of PJP and lends support for a large-scale prospective study to address this important question, the answer to which will have an immediate clinical impact. For example, if results similar to ours are obtained in a large-scale study, one possible clinical diagnostic pathway might be to collect 2 NP swab samples, one from each nostril, in the first instance. A positive result with any swab specimen would then obviate the need to collect a lower respiratory tract specimen, narrowing the requirement for these more invasive investigations to those who test negative with both NP swab specimens.

## Supplementary Material

ofae071_Supplementary_Data

## Supplementary Data

Supplementary materials are available at *Open Forum Infectious Diseases* online. Consisting of data provided by the authors to benefit the reader, the posted materials are not copyedited and are the sole responsibility of the authors, so questions or comments should be addressed to the corresponding author.
